# Hepatic origin of increased serum TFF3 in gastric cancer bearing subjects

**DOI:** 10.1007/s10120-026-01782-3

**Published:** 2026-07-20

**Authors:** Komei Kuge, Junko Mukohyama, Yoshimi Yasukawa, Yuko Ishibashi, Hiroki Masuda, Tomohiko Yasuda, Yasukazu Ohmoto, Hideyuki Takeshima, Akihisa Matsuda, Hiroshi Yoshida, Yasuyuki Seto, James R. Goldenring, Toshikazu Ushijima, Sachiyo Nomura

**Affiliations:** 1 Department of Gastrointestinal Surgery, Graduate School of Medicine, The University of Tokyo, Tokyo, Japan; 2 Department of Gastroenterological Surgery, Nippon Medical School, Tokyo, Japan; 3 Division of Frontier Surgery, The Institute of Medical Science, The University of Tokyo, Tokyo, Japan; 4 Division of Epigenomics, Institute for Advanced Life Sciences, Hoshi University, Tokyo, Japan; 5 Department of Breast and Endocrine Surgery, Graduate School of Medicine, The University of Tokyo, Tokyo, Japan; 6 Department of Pharmacokinetics and Biopharmaceutics, Faculty of Pharmaceutical Sciences, Graduate School of Pharmaceutical Sciences, The University of Tokushima, Tokushima, Japan; 7 Section of Surgical Sciences, Epithelial Biology Center, Vanderbilt University Medical Center, Nashville, TN USA; 8 Department of Cell and Developmental Biology, Vanderbilt University, Nashville, TN USA; 9 Nashville Veterans Affairs Medical Center, Nashville, TN USA; 10 Department of Clinical Pharmaceutical Sciences, School of Pharmacy and Pharmaceutical Sciences, Hoshi University, 2-4-41 Ebara, Shinagawa-Ku, Tokyo, 142-8501 Japan; 11 Isotope Science Center, The University of Tokyo, Tokyo, Japan

**Keywords:** Gastric cancer, Trefoil factor 3, Surrogate markers, Interleukin-6, STAT3

## Abstract

**Background:**

Serum trefoil factor 3 (TFF3) has emerged as a promising biomarker for gastric cancer; however, its biological origin remains unclear.

**Methods:**

Gastric cancer was induced by *Helicobacter pylori* infection combined with N-methyl-N-nitrosourea (MNU), whereas a rat model was established using N-methyl-N’-nitro-N-nitrosoguanidine (MNNG) alone. Serum TFF levels were measured by ELISA. Organ-specific TFF3 expression, cytokine profiles, STAT3 activation, and epigenetic regulation were analyzed using RT-PCR, immunohistochemistry, multiplex assays, Western blotting, and ChIP–qPCR.

**Results:**

Serum TFF1–3 levels were significantly elevated in gastric cancer–bearing mice, with TFF3 showing robust diagnostic performance. In rats, serum TFF3 levels remained unchanged after total gastrectomy. TFF3 expression was selectively upregulated in the liver, accompanied by STAT3 activation. Gastric IL-6 expression was increased, suggesting portal-mediated signaling to the liver. ChIP–qPCR demonstrated sustained enrichment of H3K27ac at the TFF3 locus following IL-6 stimulation.

**Conclusions:**

Increased serum TFF3 in gastric cancer originates from the liver and is associated with IL-6–STAT3 signaling. Persistent elevation after gastrectomy may be explained by epigenetic activation, supporting the biological basis of TFF3 as a surrogate marker.

## Introduction

Gastric cancer is one of the most common malignancies worldwide and the fifth leading cause of cancer-related death [1]. Despite recent advances in chemotherapy, immunotherapy, and radiotherapy, the prognosis for patients with advanced disease remains poor. Consequently, strategies that enable the early detection of gastric cancer are essential for improving clinical outcomes.

In Japan, the Ministry of Health, Labour and Welfare recommends that individuals over 40 years of age undergo gastric X-ray screening with a barium meal or upper gastrointestinal endoscopy once every two years to reduce gastric cancer mortality [2]. Although advances such as transnasal endoscopy have improved patient tolerance, endoscopy remains more invasive than other screening methods and many individuals still avoid it because of procedural discomfort. The pepsinogen test, which reflects the degree of atrophic gastritis, is widely used to estimate the risk of gastric cancer; however, its sensitivity and specificity remain insufficient for reliable screening [3–5].

Trefoil factor family (TFF) proteins are small secreted molecules that play essential roles in maintaining gastrointestinal mucosal integrity by promoting epithelial protection and repair [6]. Their expression is closely linked to metaplastic and pre-neoplastic changes in the stomach, and recent comprehensive marker atlases have highlighted TFFs as key components of the gastric metaplasia–dysplasia spectrum [7]. In our previous study, we reported that a screening method using serum levels of TFF proteins (TFF1, TFF2, and TFF3) was superior to the pepsinogen test [8]. Among these markers, serum TFF3 showed the largest area under the receiver operating characteristic (ROC) curve, indicating that TFF3 is the most effective marker for gastric cancer screening. However, we also found that serum TFF3 levels remain elevated even after total gastrectomy in patients with gastric cancer, whereas serum TFF1 and TFF2 levels decrease after surgery.

In the present study, we have sought to determine the source of the elevated serum TFF3 levels associated with gastric cancer and to elucidate the mechanism underlying the persistently high serum TFF3 levels after gastric cancer resection. Clarifying the mechanism underlying this persistent elevation is important for understanding the extra-gastric regulation of TFF3 and for defining the validity and limitations of serum TFF3 as a non-invasive surrogate marker. Such insights will clarify the clinical relevance of serum TFF3 and determine how it can be reliably incorporated into gastric cancer risk assessment.

## Materials and methods

### Experimental animals

Six-week-old male Jcl:SD rats were obtained from CLEA Japan, Inc. (Tokyo, Japan) and used after 1 week of acclimation. The animals were housed in plastic cages with soft chip bedding in a room with a barrier system controlled for the light/dark cycle (12 h), ventilation (18 air changes per hour), temperature (23 ± 2 °C), and relative humidity (55 ± 5%). The cages and chip bedding were exchanged twice a week. All animals had free access to a basal diet (CRF-1; Oriental Yeast, Tokyo, Japan) and water with or without N-methyl-N*’*-nitro-N-nitrosoguanidine (MNNG). Five-week-old male C57BL/6 mice were obtained from Charles River Laboratories Japan, Inc. (Yokohama, Japan) and used after 1 week of acclimation. They were kept in the same conditions as rats and had free access to a basal diet (CRF-1; Oriental Yeast, Tokyo, Japan) and water with or without N-methyl-N-nitrosourea (MNU).

### Animal study design

At the beginning of the experiments, the rats were randomly allocated to four groups based on their body weights that were measured immediately before starting MNNG treatment. The 1st and 2nd groups, consisting of 5 rats each, received tap water as controls, and the 3rd and 4th groups, consisting of 25 rats each, received 150 ppm MNNG (Tokyo Chemical Industry, Tokyo, Japan) in drinking water using light-shielded bottles for 25 weeks (Fig. 2A). Because some animals died during the experimental period, the number of animals included in the final analyses was smaller than the number initially allocated. The diet and water were changed once and twice per week, respectively, and body weight was measured once weekly during the experimental period. The animals in the 2nd and 4th groups were subjected to total gastrectomy at 37 weeks. At 7 days after total gastrectomy, or at week 38, the animals were exsanguinated under deep anesthesia by inhalation of isoflurane and subjected to blood collection. Blood samples were collected from the heart and abdominal aorta at the time of gastrectomy and autopsy, respectively.

**Fig. 1:**
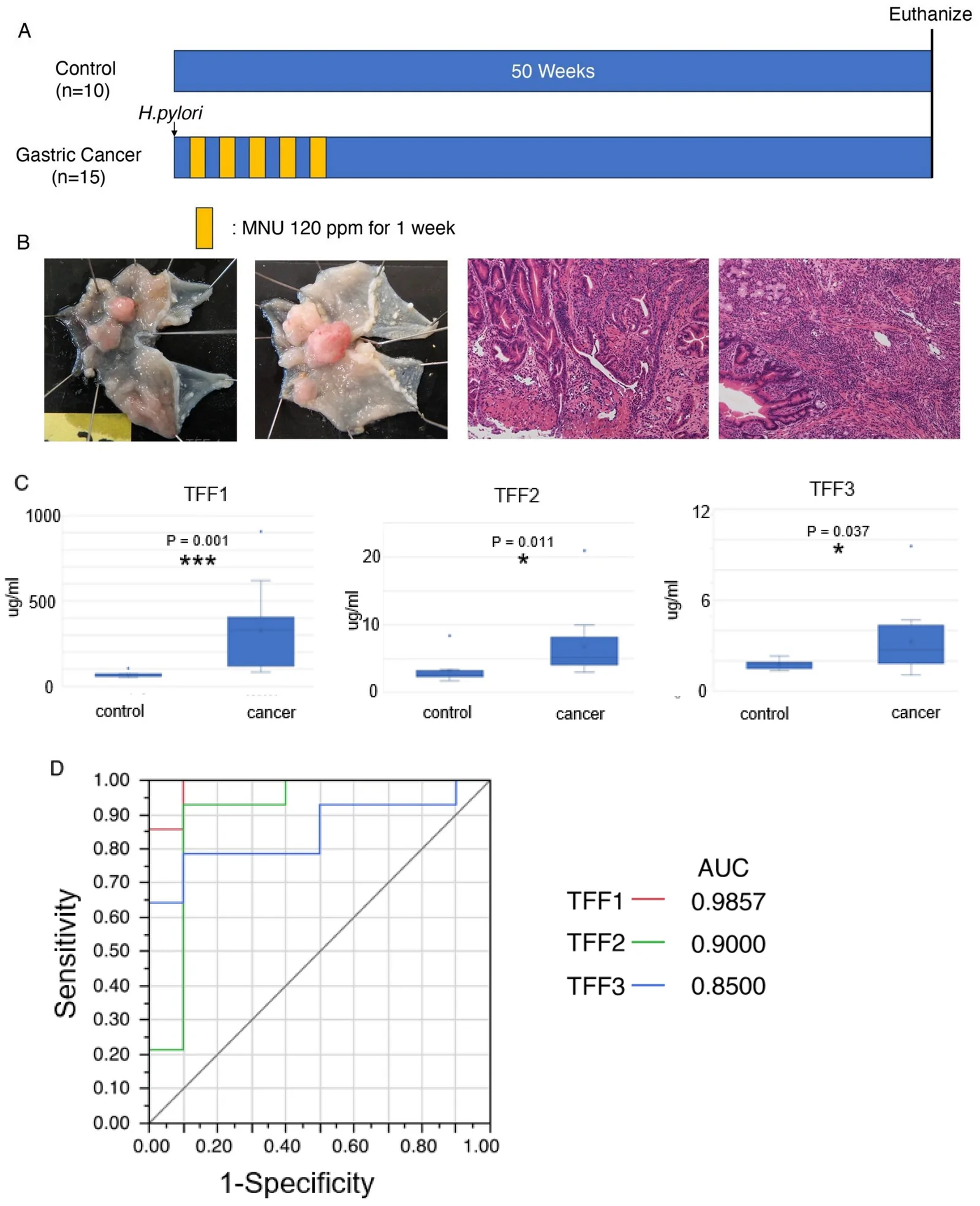
MNU-induced gastric cancer model and diagnostic performance of serum TFF1–3. **A** Experimental design and timeline. Control mice (n = 10) and MNU-treated mice (n = 15) were followed for 50 weeks; yellow blocks indicate MNU exposure (120 ppm for 1 week), and mice were euthanized at week 50. **B** Representative gross and histological images of gastric tumors in MNU-treated mice, showing differentiated and undifferentiated adenocarcinomas (hematoxylin and eosin staining). **C** Serum concentrations of TFF1, TFF2, and TFF3 in the control and gastric cancer groups. **D** Receiver operating characteristic (ROC) curves and areas under the curve (AUC) for serum TFF1, TFF2, and TFF3 for discriminating gastric cancer from controls (AUC = 0.986, 0.900, and 0.850, respectively)

The mice were randomly allocated to two groups, based on their body weights. The 1st group, consisting of 10 mice, received tap water as controls. The 2nd group, consisting of 15 mice, were infected with *Helicobacter pylori* (*H. pylori*) (SS1) (ATCC, VA, USA) in the way we described previously. One week after inoculation of H. pylori, the drinking water of the 2nd group contained 120 ppm MNU every other week for 11 weeks. The total duration of MNU administration was 5 weeks (Fig. 1A). The water containing MNU was changed every other day. The mice were exsanguinated under deep anesthesia by inhalation of isoflurane and subjected to blood collection from the heart, followed by laparotomy with excision of the stomach and other organs in 50 weeks after starting MNU. A portion of the organ was collected from brain, heart, lung, liver, spleen, kidneys, testes, pancreas, esophagus, duodenum, jejunum, ileum, bone marrow, and stomach, and stored at − 80 °C. Blood samples were collected from the heart at the time of autopsy and serum was stored at − 80 °C until ELISA measurement. The experimental design was approved by the Animal Care and Utilization Committee of the National Institute of Health Sciences, Japan and by The University of Tokyo Animal Care and Use Committee (17-P-005), and the animals were cared for in accordance with institutional guidelines.

### ELISA analysis

Plasma TFF1, TFF2 and TFF3 in mice and rats were analyzed by ELISA. Rat TFF1 and TFF2 complementary DNA (cDNA) were chemically synthesized using NCBI Reference Sequences (ratTFF1: NM_057129.2, ratTFF2: M97255, ratTFF3: NM_013042). For His-tagged *E coli* expression, rat TFF1, TFF2, and TFF3 cDNA fragments were inserted into pET-21a( +) (Novagen) vector (pET-ratTFF1-His, pET-ratTFF2-His, pET-ratTFF3-His). BL21-CodonPlus(DE3)-RIL bacteria (Stratagene, Santa Clara, CA, USA) were transformed with pET-rat TFF1-His, pET-ratTFF2-His, and pET-rat TFF3-His. The transformed bacteria were cultured in LB broth medium at 37 °C, and recombinant protein expression was induced by adding 1 mmol/L IPTG for 3 h. Bacterial pellets were harvested, and the soluble protein fractions were extracted by sonication in 0.2% Triton X-100 and 50 mmol/L Tris–HCl (pH 8.0), and recombinant rat TFFs were purified by Ni-Resin chromatography (Invitrogen, Tokyo, Japan). The recombinant rat TFFs were eluted from Ni-Resin with 0.5 mol/L imidazole, 50 mmol/L Tris–HCl (pH 8.0), and 0.5 mol/L NaCl. Each fraction from the elution was analyzed by sodium dodecyl sulfate/polyacrylamide gel electrophoresis and Western blotting. Antiserum for rat TFFs was prepared by immunizing three rabbits with 100 μg of purified rat TFFs five times every two weeks. The rat TFFs antibody was purified using a Protein A column, and its purity was confirmed by SDS-PAGE.

Purified polyclonal antibodies were coated onto 96-well microtiter plates, and the plates were blocked with 0.1% bovine serum albumin in phosphate-buffered saline. Then the blocking solution was removed, and 100 μL of assay buffer (1 mol/L NaCl, 0.1% bovine serum albumin, phosphate-buffered saline) was added to each well. Fifty microliters of the samples or standard rat TFFs were added to the wells. After incubation overnight at room temperature, the plates were washed, and diluted biotin-labeled anti-TFF polyclonal antibodies were added to each appropriate well. After incubation for 2 h, the plate was washed, and diluted horseradish peroxidase–conjugated streptavidin (Vector Laboratories, Burlingame, CA) was added to each well. After incubation for 2 h at room temperature, the plates were washed, and TMB solution (Scytek Laboratories, Inc, West Logan, UT) was added. After incubation for 10 min at room temperature, stop solution (Scytek Laboratories, Inc) was added, and the absorbance at 450 nm was measured. Concentrations of rat TFFs in the samples were calculated from the standard curves of recombinant rat TFFs. Sensitivities of TFFs were 7 pg/mL for TFF1, 30 pg/mL for TFF2, and 30 pg/mL for TFF3. Each TFF antibody reacted specifically and showed no cross-reactivity with the other TFFs.

### RT-PCR

Quantitative RT-PCR was performed on RNA extracted from organs of mice. Total RNA was extracted from organs with ISOGEN (Nippon Gene, Toyama, Japan) as the manufacturer instructed. One microgram of total RNA was mixed with 0.25 μg of random primer (Promega, Tokyo, Japan) and incubated at 70 °C for 10 min. Then first-strand buffer, dNTP mix, DTT, and Powerscript RT (Clontech, Otsu) were added and incubated at 42 °C for 90 min. The reaction was terminated by heating at 90 °C for 15 min. Quantitative PCR was performed with an Eco real-time PCR system (Illumina, Tokyo, Japan), using Thunderbird qPCR master mix with SYBR (TOYOBO) as in the manufacturer’s instructions. The PCR was performed with 40 cycles of 95 °C for 1 min, 60 °C for 30 s, and 72 °C for 20 s. The forward primer for mouse GAPDH was CGTCCCGTAGACAAAATGGT, the reverse primer for GAPDH was AATTTGCCGTGAGTGGAGTC, the forward primer for TFF3 was TATCCCAAATGTGCCCTGGT and the reverse primer for TFF3 was CTTGTGTTGGCTGTGAGGTC. PCR was performed in triplicate for each sample. Quantitation of cDNA template in each sample was determined using the ΔΔcomparative threshold cycle. TFF3 relative expression in each organ was compared between gastric cancer bearing mice and control mice using one-way ANOVA with Tukey’s test.

### Immunohistochemistry

Paraffin Section (4 μm) were deparaffinized, rehydrated, immersed in 0.3% H2O2 (#081–04215, FUJIFILM Wako Pure Chemical Corporation)/methanol (#137–01823, FUJIFILM Wako Pure Chemical Corporation, Osaka, Japan) solution for 10 min at room temperature to remove endogenous peroxidase and then antigen-retrieved in Immunosaver (Nishin EM, Tokyo, Japan) using a microwave for 20 min at 98 °C. After blocking the sections with goat serum at room temperature for 60 min, the primary antibodies were incubated on the slides overnight at 4 °C. For TFF3 staining, blocking with M.O.M. kit was added before the blocking of goat serum. Mouse antibody against TFF3 (eBioscience, San Diego, CA, USA; #14–4758, Clone 5UCLNT3, 1:1000 dilution), and Rabbit antibody against pSTAT3 (Cell Signaling Technology, Danvers, MA, USA; #9145, clone D3A7; dilution 1:200) were used as primary antibodies. The antigen–antibody complexes on the slides were visualized using VECTASTAIN Elite ABC HRP Kit and DAB Substrate Solution (Vector Laboratories, Burlingame, CA, USA; #SK-4105). Finally, cell nuclei were stained with hematoxylin (MUTO Pure Chemicals Co., Ltd., Tokyo, Japan).

### Cytokine measurement

IL-4, IL-6 and IL-13 concentrations in plasma were measured using a Bio-Plex Pro Mouse Cytokine 23-plex Assay (M60009RDPD; Bio-Rad Laboratories, Tokyo, Japan) and a Luminex MAGPIX system (Thermo Fisher Scientific, Waltham, MA) following the manufacturer’s specifications.

### Western blot

Mouse liver samples were homogenized in T-PER (Thermo Fisher Scientific, Waltham, MA, USA; #78,510) with Protease and Phosphatase inhibitor (Thermo Fisher Scientific, Waltham, MA, USA; #78,442), and total protein concentration was determined using BCA protein assays (Thermo Fisher Scientific, Waltham, MA, USA; #23,228). Proteins (50 μg per well) were loaded on 10% Mini-PROTEAN® TGX™ Precast Gels (Bio-Rad, Hercules, CA, USA; #456–1035) and transferred onto polyvinylidene fluoride membranes (Merck Millipore, Burlington, MA, USA; Immobilon-P PVDF membrane). After blocking in 5% skim milk, membranes were incubated with primary antibodies: β-actin (Thermo Fisher Scientific, Waltham, MA, USA; #PA1-16,889; dilution 1:5000) and p-STAT3 (Cell Signaling Technology, Danvers, MA, USA; #9145, clone D3A7; dilution 1:2000), followed by incubation with HRP-conjugated secondary antibodies. Detection was performed using Trident femto-ECL Western Blotting Substrate (GeneTex, Irvine, CA, USA; #GTX14689) and imaged with the MYECL Imager (Thermo Fisher Scientific, Waltham, MA, USA).

### Chromatin immunoprecipitation (ChIP) assay and ChIP-qPCR

Huh7, a human hepatocellular carcinoma cell line, was purchased from the JCRB Cell Bank (Osaka, Japan), and cells were cultured in DMEM containing 10% FBS. Cells were seeded at day 0 and treated with IL-6 at day 2 for 2 days. Cells were harvested at days 4 or 6 for ChIP assay. Chromatin was cross-linked with 1% formaldehyde for 10 min and fragmented by sonication using a Bioruptor (Cosmo Bio, Tokyo, Japan). After centrifugation, the supernatant containing fragmented chromatin was collected. Thirty micrograms (30 μg) of fragmented chromatin was incubated with 2 μg of an antibody against H3K27ac (Abcam, Cambridge, UK; #ab4729) at 4 °C overnight. The immunocomplex was collected using Dynabeads Protein A (Thermo Fisher Scientific, Waltham, MA, USA), and cross-links were reversed in the presence of 200 mM NaCl at 65 °C overnight, followed by proteinase K treatment. DNA was purified by phenol–chloroform extraction and ethanol precipitation. H3K27ac levels at TFF3 regions were analyzed by quantitative PCR using the following primers: enhancer (fwd, CTGATCTCAGCTCCTGCCAG; rev, ACTGGGCCCTGTGGAAAAC), promoter (fwd, CAGGAGTTGTGAGAGAGCCG; rev, CATGACAAGAGAGCCCCAGG), gene body (fwd, CTTCTGAGGCACCTCCAGC; rev, GAGATGAACAGTGCCTGGCA), and downstream (fwd, GGAGGCTGGAAATTCTCCCC; rev, CCCCAGGGCTCTCTAACTCT). H3K27ac and H3K4me3 levels throughout the TFF3 gene region in the Huh7 cell line were obtained from ChIP-Atlas [9, 10].

## Results

### Serum TFF1–3 accurately discriminate gastric cancer in MNU-treated mice

All 15 mice treated with MNU developed gastric cancer (Fig. 1A). The tumors were most frequently located at the fundic–pyloric border, but were also observed in the fundic and pyloric regions. Figure 1B shows representative macroscopic and microscopic features of gastric cancers in mice. Some gastric cancers were differentiated adenocarcinomas, whereas others were poorly differentiated adenocarcinomas.

Figure 1C shows the serum levels of TFF1, TFF2, and TFF3 in mice in the control group and the gastric cancer group. The serum level of TFF1 in the gastric cancer group was 328.1 ± 226.5 ng/µL, which was significantly higher than that in the control group (67.5 ± 15.4 ng/µL;* p* = 0.001). The serum level of TFF2 in the gastric cancer group was 6.82 ± 4.48 ng/µL and was also significantly higher than that in the control group (3.19 ± 1.90 ng/µL; *p* = 0.011). Similarly, the serum level of TFF3 in the gastric cancer group was 3.26 ± 2.08 ng/µL, which was significantly higher than that in the control group (1.78 ± 0.29 ng/µL; *p* = 0.037). There was no correlation between tumor weight and serum TFF3.

Figure 1D shows receiver operating characteristic (ROC) curves for serum TFF1, TFF2, and TFF3 levels to evaluate their diagnostic accuracy for gastric cancer in mice. The areas under the curve (AUCs) for serum TFF1, TFF2, and TFF3 were 0.986, 0.900, and 0.850, respectively.

### Serum TFF3 remains unchanged after total gastrectomy in a rat gastric cancer model

**Fig. 2:**
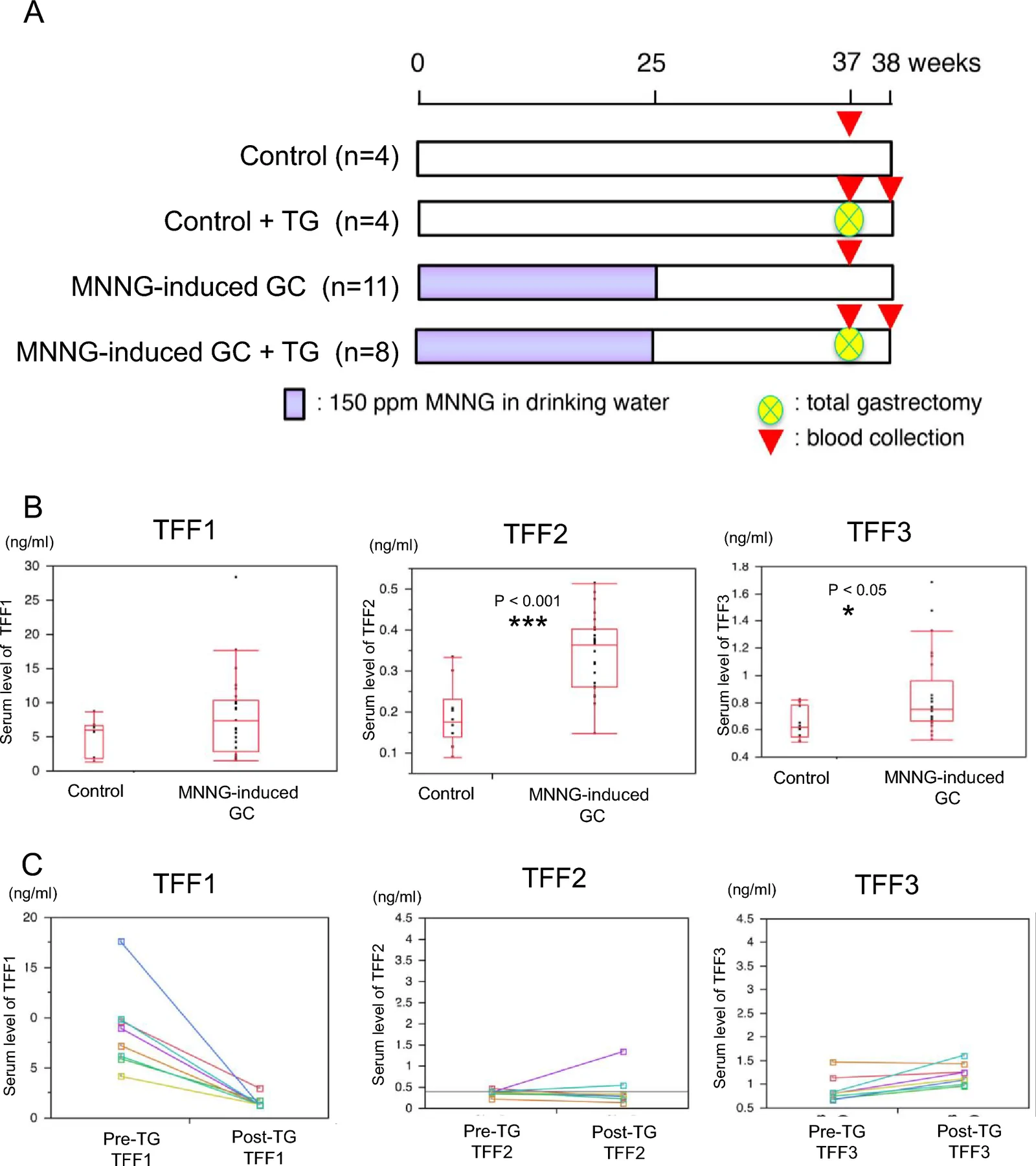
Plasma TFF3 is not reduced after total gastrectomy in an MNNG-induced rat gastric cancer model. **A** Experimental design and timeline. Rats received 150 ppm MNNG in drinking water for 25 weeks (purple). Red triangles indicate blood collection (weeks 37 and 38), and green circles indicate total gastrectomy (week 38). Group allocation and sample sizes were as follows: Control without gastrectomy (n = 4), Control with total gastrectomy (n = 4), MNNG-treated without gastrectomy (n = 11), and MNNG-treated with total gastrectomy (n = 8). **B** Preoperative plasma concentrations of TFF1, TFF2, and TFF3 in control and MNNG-treated rats. **C** Paired pre- and post-gastrectomy plasma concentrations of TFF1, TFF2, and TFF3 in rats undergoing total gastrectomy

Because mice are too small to undergo gastrectomy, we used rats to determine whether serum TFF3 levels remained elevated after total gastrectomy. Figure 2A summarizes the experimental design for the rat gastric cancer model. Rats were assigned to four groups: Control, Control + total gastrectomy, MNNG-induced gastric cancer, and MNNG-induced gastric cancer + total gastrectomy. Gastric cancer was induced by administering MNNG in drinking water for 25 weeks, followed by blood collection at weeks 37 and 38 and total gastrectomy at week 38 in the gastrectomy groups. We treated MNNG to 50 rats and 28 rats survived for 37 weeks. Tumor was developed in 19 rats out of 28 (67.9%).

Figure 2B shows the serum levels of TFF1, TFF2, and TFF3 in preoperative conditions, comparing the control group and the gastric cancer group. In rats, there was no significant difference in preoperative serum TFF1 levels between the two groups; however, preoperative serum TFF2 and TFF3 levels were significantly higher in the gastric cancer group than in the control group (TFF2, *p* < 0.001; TFF3, *p* < 0.05). After gastrectomy, serum TFF1 levels clearly decreased, despite the lack of a significant preoperative elevation in the gastric cancer group, and serum TFF2 levels showed a slight decrease. In contrast, serum TFF3 levels did not decrease after surgery, indicating the absence of a reduction in serum TFF3 levels following total gastrectomy (Fig. 2C).

### Liver-specific upregulation of TFF3 in gastric cancer–bearing mice

**Fig. 3:**
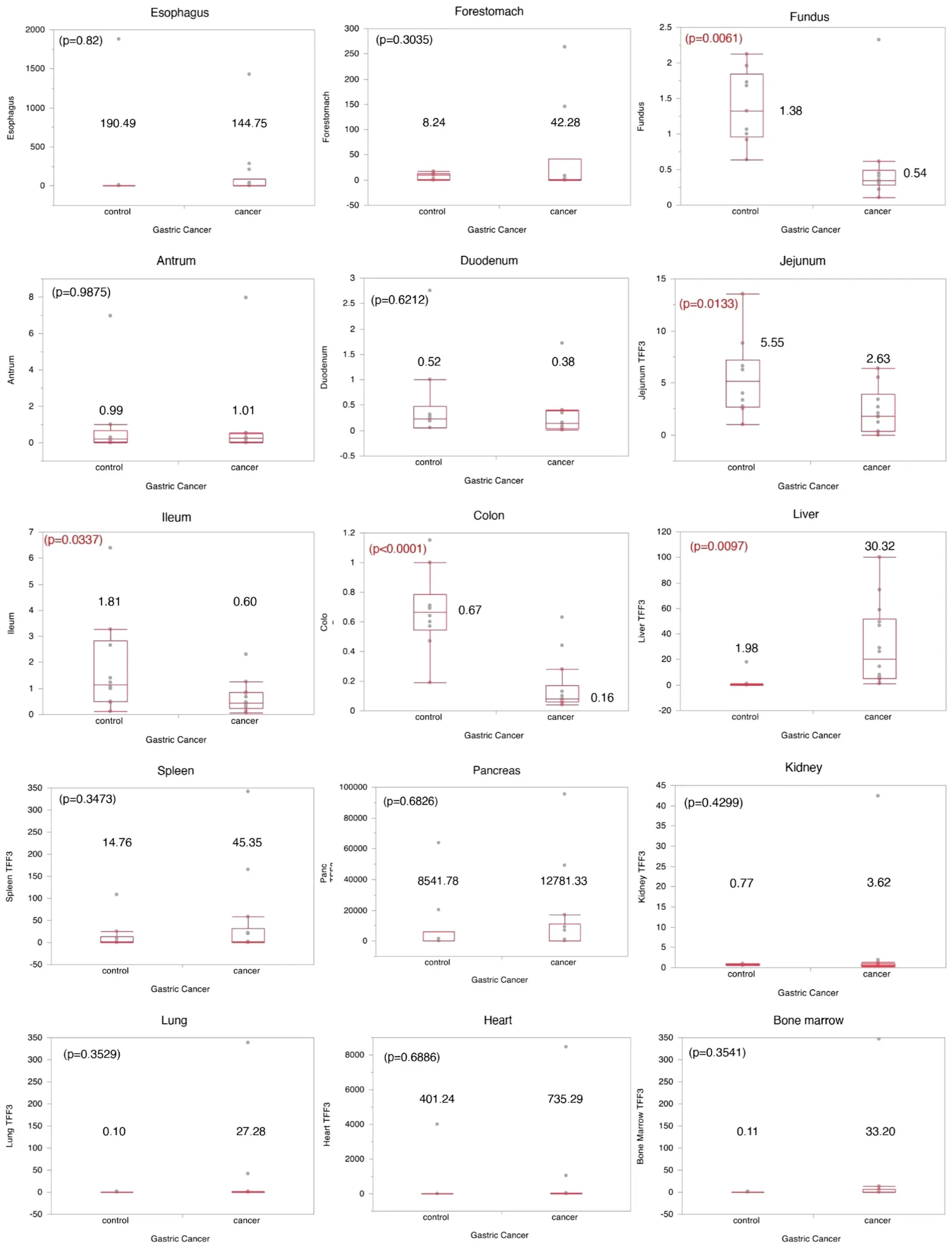
Organ distribution of TFF3 mRNA expression in control and gastric cancer–bearing mice. RT-PCR analysis of TFF3 mRNA expression in the esophagus, forestomach, fundus, antrum, duodenum, jejunum, ileum, colon, liver, spleen, pancreas, kidney, lung, heart, and bone marrow from control and gastric cancer–bearing mice. TFF3 expression was significantly increased in the liver of gastric cancer–bearing mice, whereas it was significantly decreased in the fundus, jejunum, ileum, and colon.* P* values are shown in each panel; red* p* values indicate statistically significant differences between groups

Figure 3 shows the RT-PCR results for TFF3 mRNA expression in various organs (esophagus, forestomach, fundus, antrum, duodenum, jejunum, ileum, colon, liver, spleen, pancreas, kidney, lung, heart, and bone marrow) in mice from the control group and the gastric cancer group. Among all analyzed organs, only the liver showed significantly higher TFF3 expression in gastric cancer–bearing mice compared with control mice. Interestingly, TFF3 expression was significantly lower in the gastric fundus, jejunum, ileum, and colon of gastric cancer–bearing mice. These results suggest that the liver was the major contributor to the elevated serum TFF3 levels observed in gastric cancer–bearing mice.

### Gastric IL-6 upregulation is associated with increased hepatic TFF3 in gastric cancer–bearing mice

**Fig. 4:**
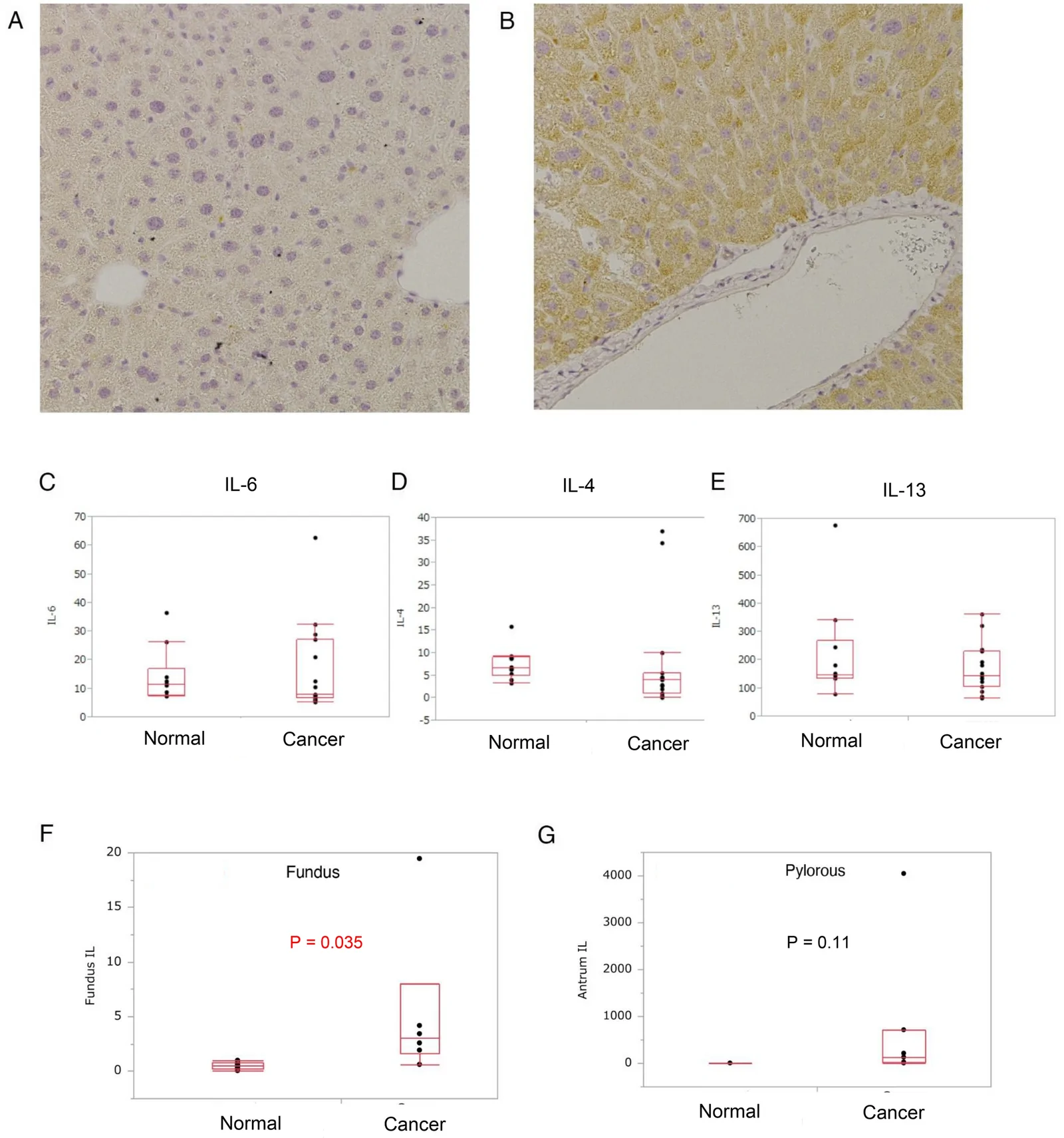
Increased hepatic TFF3 immunostaining and gastric Il6 expression in mice with gastric cancer. **A**, **B** Representative immunohistochemical staining for TFF3 in the liver from control mice (**A**) and mice with gastric cancer (**B**), showing stronger hepatic TFF3 immunostaining in mice with gastric cancer. **C**–**E** Serum levels of IL-6 (**C**), IL-4 (**D**), and IL-13 (**E**) in control mice and mice with gastric cancer. No statistically significant differences were observed between groups. **F**, **G** RT-PCR analysis of IL-6 mRNA expression in gastric tissues from control mice and mice with gastric cancer. IL-6 expression was significantly higher in the fundus (**F**), whereas no significant difference was observed in the pyloric region (**G**). P values are shown in each panel; red p values indicate statistically significant differences between groups

We performed immunohistochemical staining for TFF3 in the livers of control mice and gastric cancer–bearing mice. TFF3 showed stronger staining in the livers of gastric cancer–bearing mice, whereas only weak staining was observed in control mice (Fig. 4A, B). Because interleukin (IL)-6, IL-4, and IL-13 have been reported to induce TFF3 expression, the serum levels of these cytokines were measured. There were no significant differences in the serum levels of IL-6, IL-4, or IL-13 between the two groups, although IL-6 levels tended to be higher in gastric cancer–bearing mice (Fig. 4C–E).

However, because serum samples were collected from the heart rather than from the portal circulation between the stomach and liver, local cytokine production in the stomach might not have been adequately reflected. Therefore, we performed RT-PCR analysis of these cytokines in gastric tissues. Although there was no significant difference in IL-6 expression in the antrum, IL-6 expression in the fundus was significantly higher in gastric cancer–bearing mice than in control mice (Fig. 4F, G).

These results suggested that hepatic TFF3 production might be induced by IL-6 produced in the gastric cancer–bearing stomach and influence the liver via the portal circulation.

### Hepatic STAT3 activation in mice with gastric cancer

**Fig. 5:**
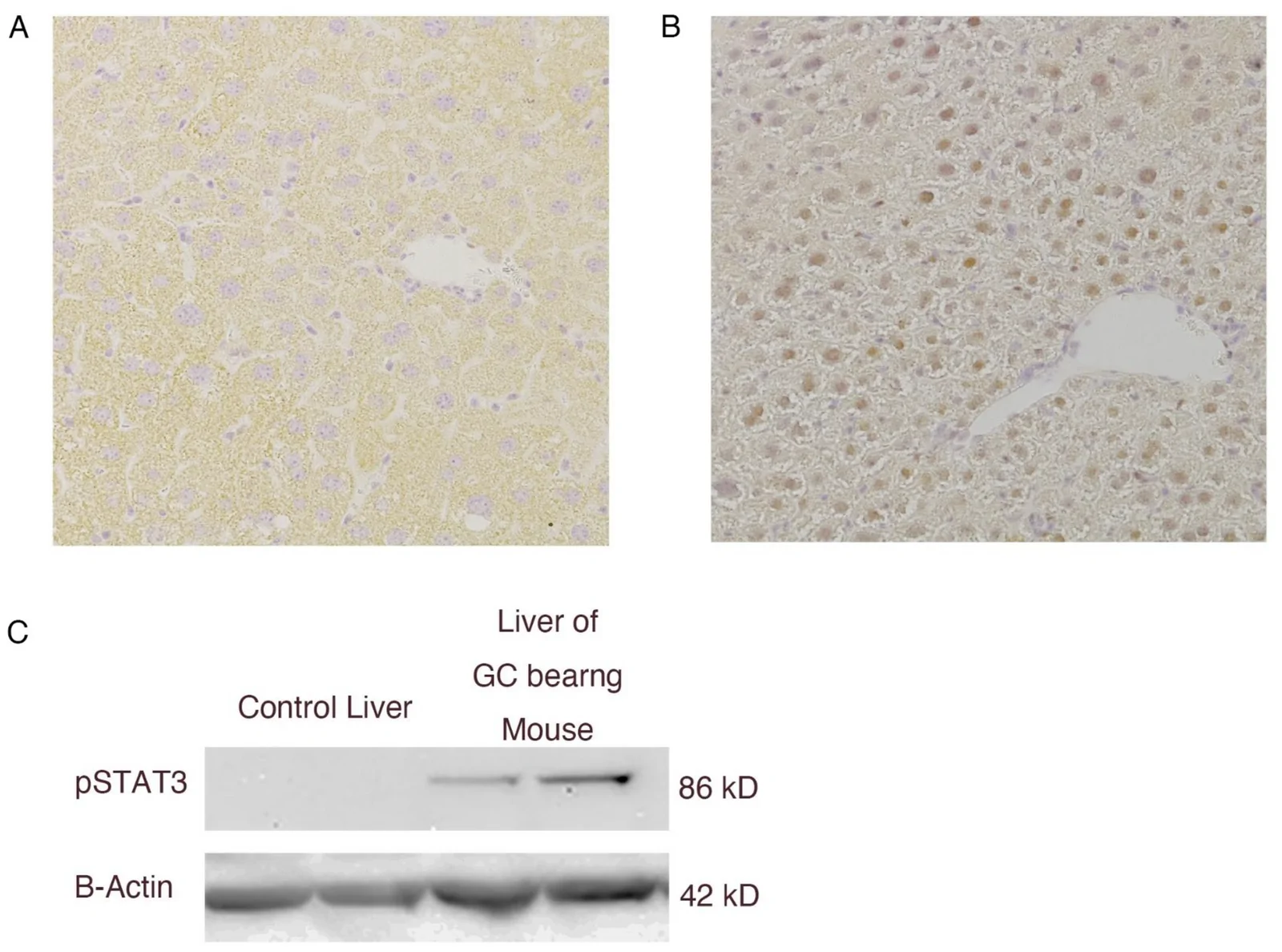
Hepatic STAT3 activation in mice with gastric cancer. **A**, **B** Representative immunohistochemical staining for phosphorylated STAT3 (pSTAT3) in the liver from control mice (**A**) and mice with gastric cancer (**B**). Nuclear pSTAT3 staining in hepatocytes was observed in mice with gastric cancer but not in control mice. **C** Western blot analysis of pSTAT3 in liver lysates from control mice and mice with gastric cancer. β-Actin was used as a loading control

Previous studies have shown that IL-6 activates STAT3 and can induce TFF3 expression in epithelial cells [11, 12]. Accordingly, to provide mechanistic support for our hypothesis that gastric IL-6 contributes to hepatic TFF3 upregulation, we assessed STAT3 activation in the liver by immunostaining for phosphorylated STAT3 (pSTAT3), the activated form that translocates to the nucleus. Nuclear pSTAT3 staining was detected in hepatocytes of gastric cancer–bearing mice (Fig. 5B), whereas pSTAT3 staining was absent in control mice (Fig. 5A). Consistently, western blot analysis also showed increased hepatic pSTAT3 levels in gastric cancer–bearing mice (Fig. 5C).

While elevated serum TFF3 levels observed in gastric cancer–bearing mice were considered to originate from the liver and to be induced by IL-6 derived from the stomach. because gastrectomy eliminates IL-6 signaling from the stomach, additional mechanisms may be required to maintain elevated serum TFF3 levels after gastrectomy. We therefore investigated whether epigenetic regulation, specifically histone modification at the TFF3 locus, could contribute to persistent TFF3 expression in hepatocytes.

**Fig. 6:**
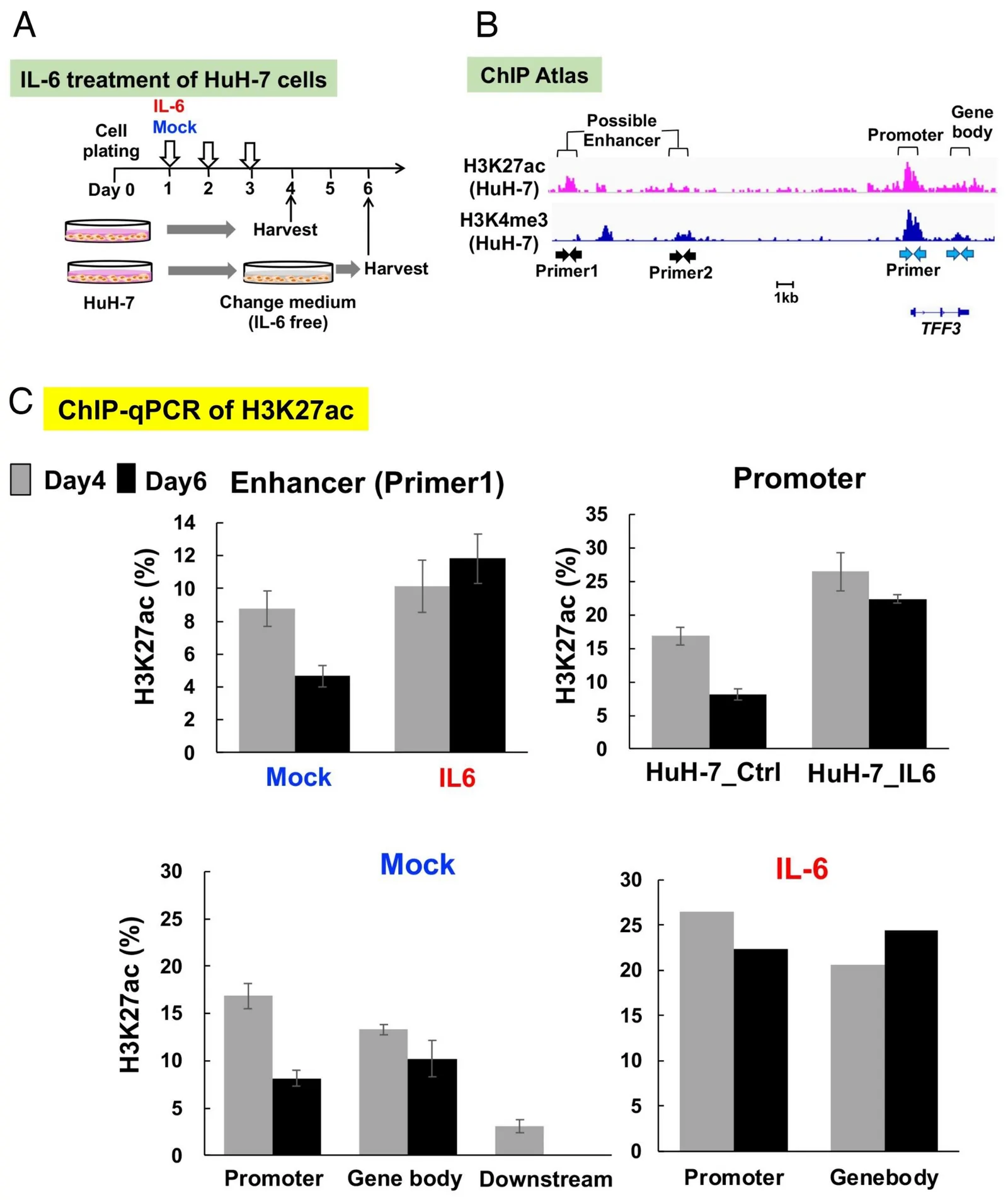
IL-6 induces sustained H3K27ac enrichment at the TFF3 locus in HuH-7 cells. **A** Experimental design for IL-6 treatment and washout in HuH-7 cells. Cells were treated with IL-6 or mock for 3 days and harvested at day 4, or cultured for an additional 2 days in IL-6–free medium and harvested at day 6. **B** ChIP-Atlas showing H3K27ac and H3K4me3 signals at the TFF3 locus in HuH-7 cells, with candidate enhancer and promoter regions and primer locations used for ChIP–qPCR. **C** ChIP–qPCR analysis of H3K27ac at the enhancer (Primer 1) and promoter regions (top) and at additional regions within the TFF3 locus (bottom). H3K27ac levels increased with IL-6 stimulation and remained elevated after IL-6 withdrawal

Histone H3 lysine 27 acetylation (H3K27ac) is a histone modification enriched at active enhancers and promoters. HuH-7 cells, a human hepatocellular carcinoma cell line, were treated with or without IL-6 for 3 days, and an additional group of cells was harvested after a 2-day cessation of IL-6 treatment. The experimental protocol is shown in Fig. 6A. The ChIP atlas profiles of H3K27ac and H3K4me3 across the TFF3 locus in HuH-7 cells were used to guide the selection of candidate regulatory regions for ChIP–qPCR (Fig. 6B). ChIP–qPCR analysis demonstrated that H3K27ac levels at the candidate enhancer, promoter, as well as at the gene body regions, of the TFF3 gene were increased by IL-6 stimulation and remained elevated even after IL-6 withdrawal, consistent with sustained epigenetic activation of the TFF3 locus. Collectively, these findings suggest that IL-6–induced epigenetic activation of the TFF3 locus may contribute to sustained elevation of circulating TFF3 levels after gastrectomy.

## Discussion

We previously reported that serum TFFs are useful biomarkers for the detection of gastric cancer [8]. Among TFF1, TFF2, and TFF3, serum TFF3 was identified as the most sensitive marker. However, serum TFF3 levels did not decrease after gastrectomy, and the origin of persistently elevated serum TFF3 remained unclear. Initially, we hypothesized that the source of TFF3 might shift before and after gastrectomy: prior to surgery, elevated TFF3 might originate from gastric cancer tissue, whereas after surgery it might be derived from the intestine due to rapid intestinal exposure to food. However, serum TFF3 levels were instead lower in patients who underwent Roux-en-Y gastric bypass for obesity compared with preoperative levels (data not shown). Based on these findings, we employed animal models of gastric cancer to investigate the origin of elevated serum TFF3 in the cancer-bearing state.

Both *H. pylori* and MNU-treated gastric cancer–bearing mice and MNNG-treated gastric cancer–bearing rats exhibited elevated serum TFF3 levels. In our previous human study, *H. pylori* infection alone did not affect serum TFF3 levels. In the present study, elevated serum TFF3 levels were also observed in tumor-bearing rat models without *H. pylori* infection. Taken together, these findings suggest that the elevation of serum TFF3 is associated with the presence of gastric cancer itself rather than with *H. pylori* infection or inflammations. At 25 weeks after MNU treatment, one mouse was euthanized to confirm the absence of gastric cancer and to measure the serum TFF3 level. The serum TFF3 concentration was 1.07 ng/mL and was not elevated. This finding further suggests that the increase in serum TFF3 was not simply induced by *H. pylori* infection plus MNU treatment, but rather required the presence of gastric cancer. In rats, serum TFF3 levels remained high even after total gastrectomy, closely mimicking the clinical findings observed in patients. Comparative analysis of TFF3 mRNA expression across multiple organs revealed that TFF3 expression was increased exclusively in the liver of gastric cancer–bearing mice. Notably, TFF3 mRNA expression was not elevated in either the fundus or antrum of cancer-bearing stomachs. These results suggest that the liver is a major source of elevated serum TFF3. Consistently, immunohistochemical analysis demonstrated higher TFF3 protein expression in the livers of gastric cancer–bearing mice.

Interestingly, TFF3 mRNA expression was reduced in the jejunum, ileum, and colon of gastric cancer–bearing animals, despite these tissues being known physiological sites of TFF3 expression. Quantification of goblet cell numbers in these tissues revealed no significant differences between control and gastric cancer–bearing mice (data not shown). The mechanism underlying the reduced intestinal TFF3 expression remains unclear; however, elevated circulating TFF3 levels may induce a form of negative feedback regulation.

To investigate the mechanisms underlying hepatic induction of TFF3, we examined known regulatory factors reported in the literature. Several studies have identified IL-6, IL-4, and IL-13 as inducers of TFF3 expression [11, 13, 14]. Although serum levels of IL-6, IL-4, and IL-13 showed no significant differences between control and gastric cancer–bearing mice, these cytokines were measured in systemic circulation. Because hepatic signaling is predominantly influenced by portal venous blood, we hypothesized that systemic measurements might underestimate local effects. Accordingly, we analyzed cytokine expression in gastric tissue by RT-PCR and found that IL-6 mRNA expression was markedly increased in both the fundus and antrum of gastric cancer–bearing stomachs. Elevated IL-6 expression in gastric cancer tissue has been previously reported in both epithelial and stromal compartments [15–17]. IL-6–mediated induction of TFF3 has been shown to occur via STAT3 phosphorylation and subsequent nuclear translocation. In the present study, STAT3 phosphorylation was confirmed by both immunohistochemistry and Western blot analysis.

Although the liver was identified as the primary source of elevated serum TFF3, gastrectomy would be expected to eliminate gastric IL-6 stimulation. Therefore, the mechanism responsible for persistently elevated serum TFF3 levels after gastrectomy remains incompletely understood. We speculate that IL-6 stimulation may induce epigenetic modifications at the TFF3 locus, resulting in sustained transcriptional activation even after removal of the initial stimulus. Indeed, H3K27ac levels at enhancer, promoter, and gene body regions of the TFF3 gene were increased following IL-6 stimulation and remained elevated after cessation of IL-6 exposure. Although the biological significance of this histone modification remains unclear, this phenomenon suggests the presence of transcriptional “memory” in gene expression programs following cancer-associated inflammation [18, 19].

Elevated serum TFF3 levels have been reported in various malignancies, including colorectal, lung, prostate, and cholangiocarcinoma [20–24]. Not all of these tumors drain into the portal circulation; however, cancers with portal venous drainage, such as colorectal, pancreatic, and cholangiocarcinoma, may share a mechanism similar to that observed in gastric cancer. TFF3 has also been reported as a promising biomarker for several malignancies, including prostate, thyroid, and breast cancers [25, 26]. In these cases, where blood flow does not pass through the portal vein, the source of circulating TFF3 is likely distinct.

TFF3 has been implicated in wound healing, epithelial protection, and inhibition of apoptosis [27–30]. TFF3 knockout mice develop normal intestinal architecture; however, following DSS-induced injury, they exhibit more severe mucosal inflammation than wild-type mice [31]. Notably, elevated serum TFF3 originating from the liver has also been reported following cerebral ischemia–reperfusion injury, where TFF3 exerts anti-apoptotic effects for cerebral cells via reduced caspase-3 activity [32]. Following this hypothesis, persistently elevated serum TFF3 derived from the liver may promote anti-apoptotic signaling in cancer cells, thereby contributing to tumor survival. However, further studies are required to elucidate the precise biological significance of sustained serum TFF3 elevation.
