# Correction: Hepatic origin of increased serum TFF3 in gastric cancer bearing subjects

**DOI:** 10.1007/s10120-026-01794-z

**Published:** 2026-08-25

**Authors:** Komei Kuge, Junko Mukohyama, Yoshimi Yasukawa, Yuko Ishibashi, Hiroki Masuda, Tomohiko Yasuda, Yasukazu Ohmoto, Hideyuki Takeshima, Akihisa Matsuda, Hiroshi Yoshida, Yasuyuki Seto, James R. Goldenring, Toshikazu Ushijima, Sachiyo Nomura

**Affiliations:** 1 Department of Gastrointestinal Surgery, Graduate School of Medicine, The University of Tokyo, Tokyo, Japan; 2 Department of Gastroenterological Surgery, Nippon Medical School, Tokyo, Japan; 3 Division of Frontier Surgery, The Institute of Medical Science, The University of Tokyo, Tokyo, Japan; 4 Division of Epigenomics, Institute for Advanced Life Sciences, Hoshi University, Tokyo, Japan; 5 Department of Breast and Endocrine Surgery, Graduate School of Medicine, The University of Tokyo, Tokyo, Japan; 6 Department of Pharmacokinetics and Biopharmaceutics, Faculty of Pharmaceutical Sciences, Graduate School of Pharmaceutical Sciences, The University of Tokushima, Tokushima, Japan; 7 Section of Surgical Sciences, Epithelial Biology Center, Vanderbilt University Medical Center, Nashville, TN USA; 8 Department of Cell and Developmental Biology, Vanderbilt University, Nashville, TN USA; 9 Nashville Veterans Affairs Medical Center, Nashville, TN USA; 10 Department of Clinical Pharmaceutical Sciences, School of Pharmacy and Pharmaceutical Sciences, Hoshi University, 2-4-41 Ebara, Shinagawa-Ku, Tokyo, 142-8501 Japan; 11 Isotope Science Center, The University of Tokyo, Tokyo, Japan

**Correction to: Gastric Cancer** **https://doi.org/10.1007/s10120-026-01782-3**

In this article the author’s name Junko Mukohyama was incorrectly written as Junko Mukoyama.

The original article has been corrected.

